# Climate change and health in North America: literature review protocol

**DOI:** 10.1186/s13643-020-01543-y

**Published:** 2021-01-04

**Authors:** Sherilee L. Harper, Ashlee Cunsolo, Amreen Babujee, Shaugn Coggins, Mauricio Domínguez Aguilar, Carlee J. Wright

**Affiliations:** 1 School of Public Health, University of Alberta, ECHA, 11405 87 Ave NW, Edmonton, AB T6G 1C9 Canada; 2 School of Arctic & Subarctic Studies, Labrador Institute of Memorial University, 219 Hamilton River Road, Stn B, PO Box 490, Happy Valley-Goose Bay, NL A0P 1E0 Canada; 3 Unidad de Ciencias Sociales, Universidad Autónoma de Yucatán, Calle 61 x 66 # 525. Col. Centro, Mérida, Yucatán México

**Keywords:** Climate Change, Human Health, Mental Health, North America, Canada, United States of America, Mexico, Protocol

## Abstract

**Background:**

Climate change is a defining issue and grand challenge for the health sector in North America. Synthesizing evidence on climate change impacts, climate-health adaptation, and climate-health mitigation is crucial for health practitioners and decision-makers to effectively understand, prepare for, and respond to climate change impacts on human health. This protocol paper outlines our process to systematically conduct a literature review to investigate the climate-health evidence base in North America.

**Methods:**

A search string will be used to search CINAHL®, Web of Science™, Scopus®, Embase® via Ovid, and MEDLINE® via Ovid aggregator databases. Articles will be screened using inclusion/exclusion criteria by two independent reviewers. First, the inclusion/exclusion criteria will be applied to article titles and abstracts, and then to the full articles. Included articles will be analyzed using quantitative and qualitative methods.

**Discussion:**

This protocol describes review methods that will be used to systematically and transparently create a database of articles published in academic journals that examine climate-health in North America.

**Supplementary Information:**

The online version contains supplementary material available at 10.1186/s13643-020-01543-y.

## Background

The direct and indirect impacts of climate change on human health continue to be observed globally, and these wide-ranging impacts are projected to continue to increase and intensify this century [1, 2]. The direct climate change effects on health include rising temperatures, which increase heat-related mortality and morbidity [3–5], and increased frequency and intensity of storms, resulting in increased injury, death, and psychological stressors [2, 6–8]. Indirect climate change impacts on health occur via altered environmental conditions, such as climate change impacts on water quality and quantity, which increase waterborne disease [9–13]; shifting ecosystems, which increase the risk of foodborne disease [14–16], exacerbate food and nutritional security [17, 18], and change the range and distribution of vectors that cause vectorborne disease [19, 20]; and place-based connections and identities, leading to psycho-social stressors and potential increases in negative mental health outcomes and suicide [6, 8]. These wide-ranging impacts are not uniformly or equitably distributed: children, the elderly, those with pre-existing health conditions, those experiencing lower socio-economic conditions, women, and those with close connections to and reliance upon the local environment (e.g. Indigenous Peoples, farmers, fishers) often experience higher burdens of climate-health impacts [1, 2, 21]. Indeed, climate change impacts on human health not only are dependent on exposure to climatic and environmental changes, but also depend on climate change sensitivity and adaptive capacity—both of which are underpinned by the social determinants of health [1, 22, 23].

The inherent complexity, great magnitude, and widespread, inequitable, and intersectional distribution of climate change impacts on health present an urgent and grand challenge for the health sector this century [2, 24, 25]. Climate-health research and evidence is critical for informing effective, equitable, and timely adaptation responses and strategies. For instance, research continues to inform local to international climate change and health vulnerability and adaptation assessments [26]. However, to create evidence-based climate-health adaptation strategies, health practitioners, researchers, and policy makers must sift and sort through vast and often unmanageable amounts of information. Indeed, the global climate-health evidence base has seen exponential growth in recent years, with tens of thousands of articles published globally this century [22, 25, 27, 28]. Even when resources are available to parse through the evidence base, the available research evidence may not be locally pertinent to decision-makers, may provide poor quality of evidence, may exclude factors important to decision-makers, may overlook temporal and geographical scales over which decision-makers have impact, and/or may not produce information in a timely manner [29–37].

Literature reviews that utilize systematic methods present a tool to efficiently and effectively integrate climate-health information and provide data to support evidence-based decision-making. Furthermore, literature reviews that use systematic methods are replicable and transparent, reduce bias, and are ultimately intended to improve reliability and accuracy of conclusions. As such, systematic approaches to identify, explore, evaluate, and synthesize literature separates insignificant, less rigorous, or redundant literature from the critical and noteworthy studies that are worthy of exploration and consideration [38]. As such, a systematic approach to synthesizing the climate-health literature provides invaluable information and adds value to the climate-health evidence base from which decision-makers can draw from. Therefore, we aim to systematically and transparently create a database of articles published in academic journals that examine climate-health in North America. As such, we outline our protocol that will be used to systematically identify and characterize literature at the climate-health nexus in North America.

## Methods

This protocol was designed in accordance with the Preferred Reporting Items for Systematic Review and Meta-Analyses (PRISMA) Guidelines [39, 40] and presented in accordance with the PRISMA-P checklist.

### Research questions

Research on climate change and human health encompasses a diverse range of health outcomes, climate change exposures, populations, and study designs. Given the breadth and depth of information needed by health practitioners and decision-makers, a variety of research questions will be examined (Table 1).

**Table 1:** Examples of research questions guiding the climate change and health in North America literature review

Example questions		
1.	What types of published literature exist on climate change and health in North America?	• Primary research articles • Review articles using systematic methods
2.	What are the North American publication trends over time?	• Year of article publication
3.	What is the geographical distribution of research and data in North America?	• Canada • United States of America (USA) • Mexico • Multinational, including North America
4.	What types of climate-health data are being assessed in the literature? What is the nature of climate-health literature and state-of-knowledge for different health outcomes?	• Weather data (e.g. temperature, UV, precipitation) • Climatic hazard data (e.g. hurricanes, wildfires, heat events, air quality) • Health data (e.g. heat morbidity and/or mortality, respiratory illness, vectorborne diseases) • Social characteristics as they relate to impacts on climate-sensitive health outcomes (e.g. gender, income, education, ethnicity)
5.	What aspects of climate change are being focused on in North America?	• Climate change impacts • Climate change adaptation • Climate change mitigation
6.	What research methodologies are being used?	• Qualitative research/quantitative research/mixed qualitative and quantitative research • Inclusion of future climate projections
7.	What are key climate-health risks in North America?	• Intersection of climatic variables, health outcomes, and social characteristics
8.	What climate-health adaptation strategies are effective in North America, for whom, under what conditions, and why?	• Climate change adaptation

### Search strategy

The search strategy, including the search string development and selection of databases, was developed in consultation with a research librarian and members of the research team (SLH, AC, and MDA). The search string contains terms related to climate change [41, 42], human health outcomes [1, 25, 43, 44], and study location (Table 2). Given the interdisciplinary nature of the climate-health nexus and to ensure that our search is comprehensive, the search string will be used to search five academic databases:

1. CINAHL® will be searched to capture unique literature not found in other databases on common disease and injury conditions, as well as other health topics;
2. Web of Science™ will be searched to capture a wide range of multi-disciplinary literature;
3. Scopus® will be searched to capture literature related to medicine, technology, science, and social sciences;
4. Embase® via Ovid will be searched to capture a vast range of biomedical sciences journals; and
5. MEDLINE® via Ovid will be searched to capture literature on biomedical and health sciences.

**Table 2:** Search strategy for CINAHL® to identify published articles reporting on climate change impacts on human health in North America published after 2013 (see Appendix 1 for search strategies for Web of Science™, Scopus®, Embase® via Ovid, and MEDLINE® via Ovid)

Component	Search term
North America	Canada OR “North America” OR “United States” OR “United States of America” OR USA OR Mexico OR “United Mexican States” OR “British Columbia” OR Alberta OR Manitoba OR Saskatchewan OR Ontario OR Quebec OR “Prince Edward Island” OR PEI OR “Nova Scotia” OR “New Brunswick” OR Newfoundland OR Labrador OR Yukon OR Nunavut OR “Northwest Territories” OR NWT OR Alabama OR Alaska OR Arizona OR Arkansas OR California OR Colorado OR Connecticut OR Delaware OR Florida OR Georgia OR Hawaii OR Idaho OR Illinois OR Indiana OR Iowa OR Kansas OR Kentucky OR Louisiana OR Maine OR Maryland OR Massachusetts OR Michigan OR Minnesota OR Mississippi OR Missouri OR Montana OR Nebraska OR Nevada OR “New Hampshire” OR “New Jersey” OR “New Mexico” OR “New York” OR “North Carolina” OR “North Dakota” OR Ohio OR Oklahoma OR Oregon OR Pennsylvania OR “Rhode Island” OR “South Carolina” OR “South Dakota” OR Tennessee OR Texas OR Utah OR Vermont OR Virginia OR Washington OR “West Virginia” OR Wisconsin OR Wyoming OR Aguascalientes OR “Baja California” OR Campeche OR Chiapas OR Chihuahua OR Coahuila OR Colima OR Durango OR Guanajuato OR Guerrero OR Hidalgo OR Jalisco OR México OR Michoacán OR Morelos OR Nayarit OR “Nuevo León” OR Oaxaca OR Puebla OR Querétaro OR “Quintana Roo” OR “San Luis Potosí” OR Sinaloa OR Sonora OR Tabasco OR Tamaulipas OR Tlaxcala OR Veracruz OR Yucatán OR Zacatecas AND
Climate change	“climate change” OR weather OR “atmospheric pressure” OR “climatic change” OR “global warming” OR “environmental change” OR “climate disaster” OR “greenhouse effect” OR “climate variability” OR “climatic variability” OR “carbon emission” OR cold OR cool OR cooling OR heat OR humid* OR ice OR precipitation OR rain* OR season* OR snow* OR storm OR temperature OR warm OR warming OR wind OR “ultraviolet radiation” OR UV AND
Human health	health OR disease* OR morbidity OR mortality OR wellbeing OR illness* OR wellness OR infect* OR death OR injur* OR mental* OR emotion*

No language restrictions will be placed on the search. Date restrictions will be applied to capture literature published on or after 01 January 2013, in order to capture literature published after the Intergovernmental Panel on Climate Change (IPCC) Fifth Assessment Report (which assessed literature accepted for publication prior to 31 August 2013). An initial test search was conducted on June 10, 2019, and updated on February 14, 2020; however, the search will be updated to include literature published within the most recent full calendar year prior to publication.

To explore the sensitivity of our search and capture any missed articles, (1) a snowball search will be conducted on the reference lists of all the literature that meet the inclusion criteria and (2) a hand search of three relevant disciplinary journals will be conducted:

1. *Environmental Health Perspectives*, an open access peer-reviewed journal that is a leading disciplinary journal within environmental health sciences;
2. *The Lancet*, a peer-reviewed journal that is the leading disciplinary journal within public health sciences; and
3. *Climatic Change*, a peer-reviewed journal covering cross-disciplinary literature that is a leading disciplinary journal for climate change research.

Citations will be downloaded from the databases and uploaded into Mendeley™ reference management software to facilitate reference management, article retrieval, and removal of duplicate citations. Then, de-duplicated citations will be uploaded into DistillerSR® to facilitate screening.

### Article selection

#### Inclusion and exclusion criteria

**Fig. 1:**
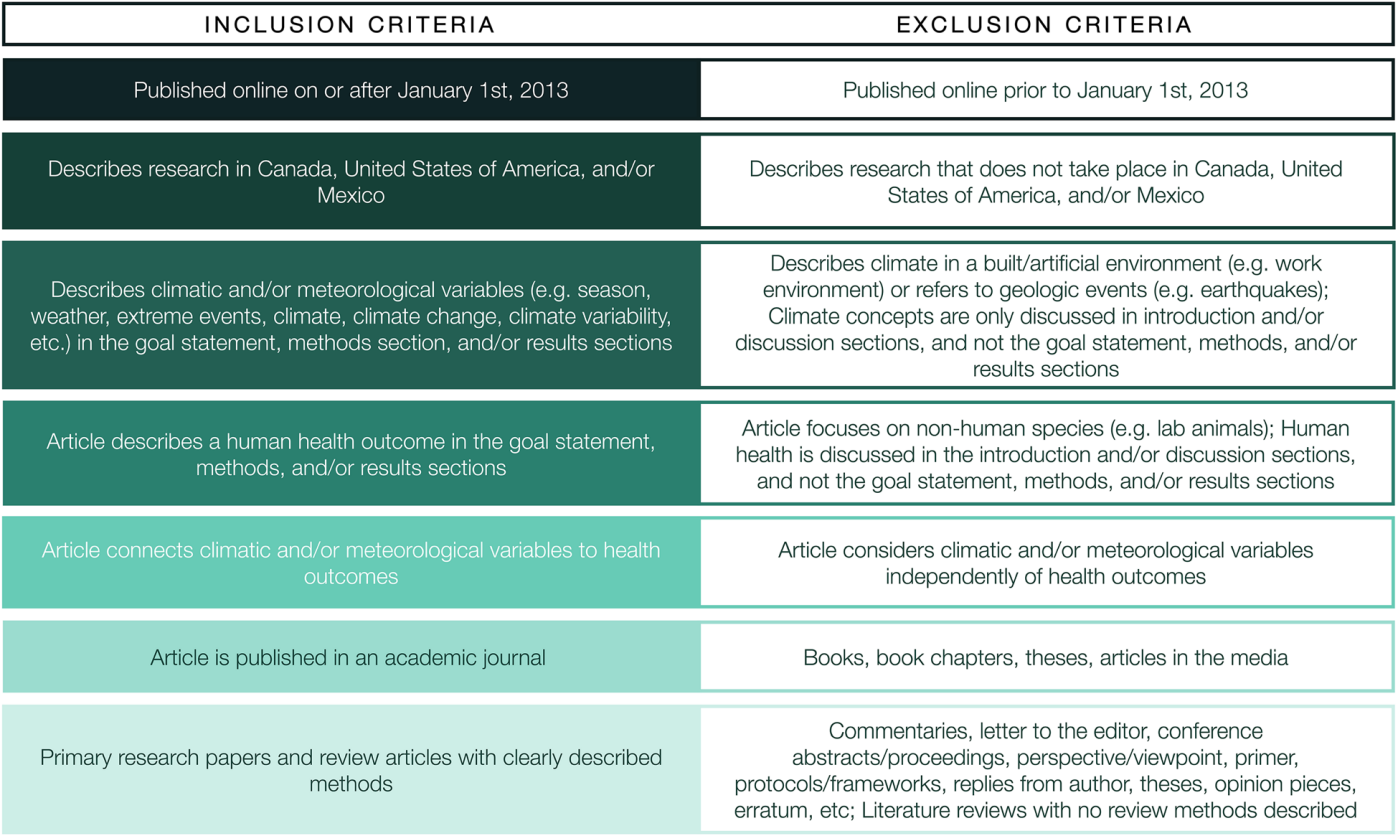
Inclusion and exclusion criteria to review climate change and health literature in North America

To be included, articles must evaluate or examine the intersection of climate change and human health in North America (Fig. 1). Health is defined to include physical, mental, emotional, and social health and wellness [1, 25, 43, 44] (Fig. 1). This broad definition will be used to examine the nuanced and complex direct and indirect impacts of climate change on human health. To examine the depth and breadth of climate change impacts on health, climate change contexts are defined to include seasonality, weather parameters, extreme weather events, climate, climate change, climate variability, and climate hazards [41, 42] (Fig. 1). However, articles that discuss climate in terms of indoor work environments, non-climate hazards due to geologic events (e.g. earthquakes), and non-anthropogenic climate change (e.g. due to volcanic eruptions) will be excluded. This broad definition of climate change contexts will be used in order to examine the wide range and complexity of climate change impacts on human health. To be included, articles need to explicitly link health outcomes to climate change in the goal statement, methods section, and/or results section of the article. Therefore, articles that discuss both human health and climate change—but do not link the two together—will be excluded. The climate-health research has to take place in North America to be included. North America is defined to include Canada, the USA, and Mexico in order to be consistent with the IPCC geographical classifications; that is, in the Fifth Assessment Report, the IPCC began confining North America to include Canada, Mexico, and the USA [45] (Fig. 1). Articles published in any language will be eligible for inclusion. Articles need to be published online on or after 01 January 2013 to be included. No restrictions will be placed on population type (i.e. all human studies will be eligible for inclusion).

#### Level 1 screening

The title and abstract of each citation will be examined for relevance. A stacked questionnaire will be used to screen the titles and abstracts; that is, when a criterion is not met, the subsequent criteria will not be assessed. When all inclusion criteria are met and/or it is unclear whether or not an inclusion criterion is met (e.g. “unsure”), the article will proceed to Level 2 screening. If the article meets any exclusion criteria, it will not proceed to Level 2 screening. Level 1 screening will be completed by two independent reviewers, who will meet to resolve any conflicts via discussion. The level of agreement between reviewers will be evaluated by dividing the total number of conflicts by the total number of articles screened for Level 1.

#### Level 2 screening

**Fig. 2:**
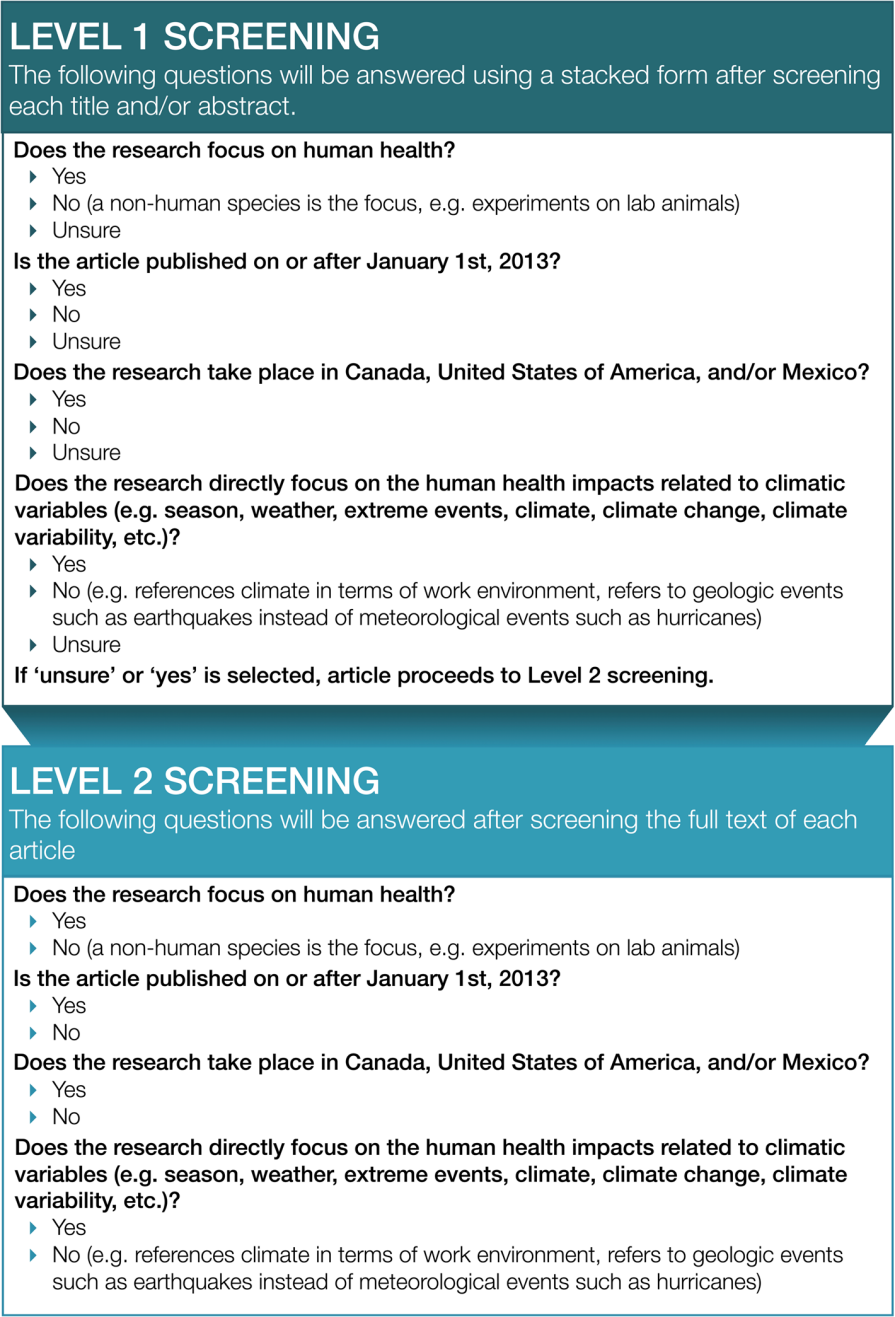
Flow chart of screening questions for the literature review on climate change and health in North America

The full text of all potentially relevant articles will be screened for relevance. A stacked questionnaire will also be used to screen the full texts. In Level 2 screening, only articles that meet all the inclusion criteria will be included in the review (i.e. “unsure” will not be an option). Level 2 screening will be completed by two independent reviewers, who will meet to resolve any conflicts via discussion. The level of agreement between reviewers will be evaluated by dividing the total number of conflicts by the total number of articles screened for Level 2 (Fig. 2).

### Data extraction and analysis

A data extraction form will be created in DistillerSR® (Appendix 2) and will be tested by three data extractors on a sample of articles to allow for calibration on the extraction process (i.e. 5% of articles if greater than 50 articles, 10% of articles if less than or equal to 50 articles). After completing the calibration process, the form will be adapted based on feedback from the extractors to improve usability and accuracy. The data extractors will then use the data extraction form to complete data extraction. Reviewers will meet regularly to discuss and resolve any further issues in data extraction, in order to ensure the data extraction process remains consistent across reviewers.

Data will be extracted from original research papers (i.e. articles containing data collection and analysis) and review articles that reported a systematic methodology. This data extraction will focus on study characteristics, including the country that the data were collected in, focus of the study (i.e. climate change impact, adaptation, and/or mitigation), weather variables, climatic hazards, health outcomes, social characteristics, and future projections. The categories within each study characteristic will not be mutually exclusive, allowing more than one response/category to be selected under each study characteristic. For the country of study, Canada, the USA, and/or Mexico will be selected if the article describes data collection in each country respectively. Non-North American regions will be selected if the article not only collects data external to North America, but also includes data collection within Canada, the USA, and/or Mexico. For the study focus, data will be extracted on whether the article focuses on climate change impacts, adaptation, and/or mitigation within the goals, methods, and/or results sections of the article. Temperature, precipitation, and/or UV radiation will be selected for weather variables if the article utilizes these data in the goal, methods, and/or results sections. Data will be extracted on the following climatic hazards if the article addresses them in the goal, methods, and/or results sections: heat events (e.g. extreme heat, heat waves), cold events (e.g. extreme cold, winter storms), air quality (e.g. pollution, parts per million (PPM) data, greenhouse gas emissions), droughts, flooding, wildfires, hurricanes, wildlife changes (including changes in disease vectors such as ticks or mosquitos), vegetation changes (including changes in pollen), freshwater (including drinking water), ocean conditions (including sea level rise and ocean acidity/salinity/temperature changes), ice extent/stability/duration (including sea ice and freshwater ice), coastal erosion, permafrost changes, and/or environmental hazards (e.g. exposure to sewage, reduced crop productivity).

Data will be extracted on the following health outcomes if the article focuses on them within the goal, methods, and/or results sections: heat-related morbidity and/or mortality, respiratory outcomes (including asthma, chronic obstructive pulmonary disease), cardiovascular outcomes (including heart attacks or stroke), urinary outcomes (e.g. urinary tract infections, renal failure), dermatologic concerns, mental health and wellness (e.g. suicide, emotional health), fetal health/birth outcomes and/or maternal health, cold exposure, allergies, nutrition (including nutrient deficiency), waterborne disease, foodborne disease, vectorborne disease, injuries (including accidents), and general morbidity and/or mortality. Data on the following social characteristics will also be extracted from the articles if they are included in the goal, methods, and/or results sections of the article: access to healthcare, sex and/or gender, age, income, livelihood (including data on employment, occupation), ethnicity, culture, Indigenous Peoples, rural/remote communities (“rural”, “remote”, or similar terminology must be explicitly mentioned), urban communities (“urban”, “city”, “metropolitan”, or similar terminology must be explicitly used), coastal communities (use of “coastal”, or similar terms must be explicitly mentioned), residence location (zipcode/postal code, neighbourhood, etc.), level of education, and housing (e.g. data on size, age, number of windows, air conditioning). Finally, data will be collected on future projections, including projections that employ qualitative and/or quantitative methods that are included in the goal, methods, and/or results sections of the article.

Descriptive statistics and regression modelling will be used to examine publication trends. Data will be visualized through the use of maps, graphs, and other visualization techniques as appropriate. To enable replicability and transparency, a PRISMA flowchart will be created to illustrate the article selection process and reasons for exclusion. Additionally, qualitative thematic analyses will be conducted. These analyses will utilize constant-comparative approaches to identify patterns across articles through the identification, development, and refinement of codes and themes. Article excerpts will be grouped under thematic categories in order to explore connections in article characteristics, methodologies, and findings.

Quality appraisal of studies included in the systematic scoping review will be performed using a framework based on the Mixed Methods Appraisal Tool (MMAT) [46] and the Confidence in the Evidence from Reviews of Qualitative Research (CERQual) tool [47]. This will enable appraisal of evidence in reviews that contain qualitative, quantitative, and mixed methods studies, as well as appraisal of methodological limitations in included qualitative studies. These tools may be adapted to include additional questions as required in order to fit the scope and objectives of the review. A minimum of two reviewers will independently appraise the included articles and discuss judgements as needed. The findings will be made available as supplementary material for the review.

## Discussion

Climate-health literature reviews using systematic methods will be increasingly critical in the health sector, given the depth and breadth of the growing body of climate change and health literature, as well as the urgent need for evidence to inform climate-health adaptation and mitigation strategies. To support and encourage the systematic and transparent identification and synthesis of climate-health information, this protocol describes our approach to systematically and transparently create a database of articles published in academic journals that examine climate-health in North America.

### Supplementary Information

**Additional file 1.**

## Data Availability

Not applicable.

## Appendix 1

Search strategy for CINAHL®, Web of Science™, Scopus®, Embase® via Ovid, and MEDLINE® via Ovid.

**Table:**

Search String	Search Limiters
CINAHL®	
(Canada OR “North America” OR “United States” OR “United States of America” OR USA OR Mexico OR “United Mexican States” OR “British Columbia” OR Alberta OR Manitoba OR Saskatchewan OR Ontario OR Quebec OR “Prince Edward Island” OR PEI OR “Nova Scotia” OR “New Brunswick” OR Newfoundland OR Labrador OR Yukon OR Nunavut OR “Northwest Territories” OR NWT OR Alabama OR Alaska OR Arizona OR Arkansas OR California OR Colorado OR Connecticut OR Delaware OR Florida OR Georgia OR Hawaii OR Idaho OR Illinois OR Indiana OR Iowa OR Kansas OR Kentucky OR Louisiana OR Maine OR Maryland OR Massachusetts OR Michigan OR Minnesota OR Mississippi OR Missouri OR Montana OR Nebraska OR Nevada OR “New Hampshire” OR “New Jersey” OR “New Mexico” OR “New York” OR “North Carolina” OR “North Dakota” OR Ohio OR Oklahoma OR Oregon OR Pennsylvania OR “Rhode Island” OR “South Carolina” OR “South Dakota” OR Tennessee OR Texas OR Utah OR Vermont OR Virginia OR Washington OR “West Virginia” OR Wisconsin OR Wyoming OR Aguascalientes OR “Baja California” OR Campeche OR Chiapas OR Chihuahua OR Coahuila OR Colima OR Durango OR Guanajuato OR Guerrero OR Hidalgo OR Jalisco OR México OR Michoacán OR Morelos OR Nayarit OR “Nuevo León” OR Oaxaca OR Puebla OR Querétaro OR “Quintana Roo” OR “San Luis Potosí” OR Sinaloa OR Sonora OR Tabasco OR Tamaulipas OR Tlaxcala OR Veracruz OR Yucatán OR Zacatecas) AND (“climate change” OR weather OR “atmospheric pressure” OR “climatic change” OR “global warming” OR “environmental change” OR “climate disaster” OR “greenhouse effect” OR “climate variability” OR “climatic variability” OR “carbon emission” OR cold OR cool OR cooling OR heat OR humid* OR ice OR precipitation OR rain* OR season* OR snow* OR storm OR temperature OR warm OR warming OR wind OR “ultraviolet radiation” OR UV) AND (health OR disease* OR morbidity OR mortality OR wellbeing OR illness* OR wellness OR infect* OR death OR injur* OR mental* OR emotion*)	Publication date: January 2013–December 2019 Filtered by: Human Countries: USA, Canada, Mexico
Web of Science™	
TOPIC: (Canada OR “North America” OR “United States” OR “United States of America” OR USA OR Mexico OR “United Mexican States” OR “British Columbia” OR Alberta OR Manitoba OR Saskatchewan OR Ontario OR Quebec OR “Prince Edward Island” OR PEI OR “Nova Scotia” OR “New Brunswick” OR Newfoundland OR Labrador OR Yukon OR Nunavut OR “Northwest Territories” OR NWT OR Alabama OR Alaska OR Arizona OR Arkansas OR California OR Colorado OR Connecticut OR Delaware OR Florida OR Georgia OR Hawaii OR Idaho OR Illinois OR Indiana OR Iowa OR Kansas OR Kentucky OR Louisiana OR Maine OR Maryland OR Massachusetts OR Michigan OR Minnesota OR Mississippi OR Missouri OR Montana OR Nebraska OR Nevada OR “New Hampshire” OR “New Jersey” OR “New Mexico” OR “New York” OR “North Carolina” OR “North Dakota” OR Ohio OR Oklahoma OR Oregon OR Pennsylvania OR “Rhode Island” OR “South Carolina” OR “South Dakota” OR Tennessee OR Texas OR Utah OR Vermont OR Virginia OR Washington OR “West Virginia” OR Wisconsin OR Wyoming OR Aguascalientes OR “Baja California” OR Campeche OR Chiapas OR Chihuahua OR Coahuila OR Colima OR Durango OR Guanajuato OR Guerrero OR Hidalgo OR Jalisco OR México OR Michoacán OR Morelos OR Nayarit OR “Nuevo León” OR Oaxaca OR Puebla OR Querétaro OR “Quintana Roo” OR “San Luis Potosí” OR Sinaloa OR Sonora OR Tabasco OR Tamaulipas OR Tlaxcala OR Veracruz OR Yucatán OR Zacatecas) AND TOPIC: (“climate change” OR weather OR “atmospheric pressure” OR “climatic change” OR “global warming” OR “environmental change” OR “climate disaster” OR “greenhouse effect” OR “climate variability” OR “climatic variability” OR “carbon emission” OR cold OR cool OR cooling OR heat OR humid* OR ice OR precipitation OR rain* OR season* OR snow* OR storm OR temperature OR warm OR warming OR wind OR “ultraviolet radiation” OR UV) AND TOPIC: (health OR disease* OR morbidity OR mortality OR wellbeing OR illness* OR wellness OR infect* OR death OR injur* OR mental* OR emotion*)	Publication date: 2013–2019 Countries: USA, Canada, Mexico
Scopus®	
(TITLE-ABS-KEY (canada OR “North America” OR “United States” OR “United States of America” OR usa OR mexico OR “United Mexican States” OR “British Columbia” OR alberta OR manitoba OR saskatchewan OR ontario OR quebec OR “Prince Edward Island” OR pei OR “Nova Scotia” OR “New Brunswick” OR newfoundland OR labrador OR yukon OR nunavut OR “Northwest Territories” OR nwt OR alabama OR alaska OR arizona OR arkansas OR california OR colorado OR connecticut OR delaware OR florida OR georgia OR hawaii OR idaho OR illinois OR indiana OR iowa OR kansas OR kentucky OR louisiana OR maine OR maryland OR massachusetts OR michigan OR minnesota OR mississippi OR missouri OR montana OR nebraska OR nevada OR “New Hampshire” OR “New Jersey” OR “New Mexico” OR “New York” OR “North Carolina” OR “North Dakota” OR ohio OR oklahoma OR oregon OR pennsylvania OR “Rhode Island” OR “South Carolina” OR “South Dakota” OR tennessee OR texas OR utah OR vermont OR virginia OR washington OR “West Virginia” OR wisconsin OR wyoming OR aguascalientes OR “Baja California” OR campeche OR chiapas OR chihuahua OR coahuila OR colima OR durango OR guanajuato OR guerrero OR hidalgo OR jalisco OR méxico OR michoacán OR morelos OR nayarit OR “Nuevo León” OR oaxaca OR puebla OR querétaro OR “Quintana Roo” OR “San Luis Potosí” OR sinaloa OR sonora OR tabasco OR tamaulipas OR tlaxcala OR veracruz OR yucatán OR zacatecas)) AND (TITLE-ABS-KEY (“climate change” OR weather OR “atmospheric pressure” OR “climatic change” OR “global warming” OR “environmental change” OR “climate disaster” OR “greenhouse effect” OR “climate variability” OR “climatic variability” OR “carbon emission” OR cold OR cool OR cooling OR heat OR humid* OR ice OR precipitation OR rain* OR season* OR snow* OR storm OR temperature OR warm OR warming OR wind OR “ultraviolet radiation” OR uv)) AND (TITLE-ABS-KEY (health OR disease* OR morbidity OR mortality OR wellbeing OR illness* OR wellness OR infect* OR death OR injur* OR mental* OR emotion*)) AND (LIMIT-TO (AFFILCOUNTRY, “United States”) OR LIMIT-TO (AFFILCOUNTRY, “Canada”) OR LIMIT-TO (AFFILCOUNTRY, “Mexico”)) AND (LIMIT-TO (PUBYEAR, 2019) OR LIMIT-TO (PUBYEAR, 2018) OR LIMIT-TO (PUBYEAR, 2017) OR LIMIT-TO (PUBYEAR, 2016) OR LIMIT-TO (PUBYEAR, 2015) OR LIMIT-TO (PUBYEAR, 2014) OR LIMIT-TO (PUBYEAR, 2013)) AND (LIMIT-TO (EXACTKEYWORD, “Human”) OR LIMIT-TO (EXACTKEYWORD, “Humans”))	Publication date: 2013–2019 Filtered by: Human/Humans Countries: USA, Canada, Mexico
Embase® via Ovid	
(Canada or “North America” or “United States” or “United States of America” or USA or Mexico or “United Mexican States” or “British Columbia” or Alberta or Manitoba or Saskatchewan or Ontario or Quebec or “Prince Edward Island” or PEI or “Nova Scotia” or “New Brunswick” or Newfoundland or Labrador or Yukon or Nunavut or “Northwest Territories” or NWT or Alabama or Alaska or Arizona or Arkansas or California or Colorado or Connecticut or Delaware or Florida or Georgia or Hawaii or Idaho or Illinois or Indiana or Iowa or Kansas or Kentucky or Louisiana or Maine or Maryland or Massachusetts or Michigan or Minnesota or Mississippi or Missouri or Montana or Nebraska or Nevada or “New Hampshire” or “New Jersey” or “New Mexico” or “New York” or “North Carolina” or “North Dakota” or Ohio or Oklahoma or Oregon or Pennsylvania or “Rhode Island” or “South Carolina” or “South Dakota” or Tennessee or Texas or Utah or Vermont or Virginia or Washington or “West Virginia” or Wisconsin or Wyoming or Aguascalientes or “Baja California” or Campeche or Chiapas or Chihuahua or Coahuila or Colima or Durango or Guanajuato or Guerrero or Hidalgo or Jalisco or Mexico or Michoacan or Morelos or Nayarit or “Nuevo Leon” or Oaxaca or Puebla or Queretaro or “Quintana Roo” or “San Luis Potosi” or Sinaloa or Sonora or Tabasco or Tamaulipas or Tlaxcala or Veracruz or Yucatan or Zacatecas).mp. [mp = title, abstract, heading word, drug trade name, original title, device manufacturer, drug manufacturer, device trade name, keyword, floating subheading word, candidate term word] AND (“climate change” or weather or “atmospheric pressure” or “climatic change” or “global warming” or “environmental change” or “climate disaster” or “greenhouse effect” or “climate variability” or “climatic variability” or “carbon emission” or cold or cool or cooling or heat or humid* or ice or precipitation or rain* or season* or snow* or storm or temperature or warm or warming or wind or “ultraviolet radiation” or UV).mp. [mp = title, abstract, heading word, drug trade name, original title, device manufacturer, drug manufacturer, device trade name, keyword, floating subheading word, candidate term word] AND (health or disease* or morbidity or mortality or wellbeing or illness* or wellness or infect* or death or injur* or mental* or emotion*).mp. [mp = title, abstract, heading word, drug trade name, original title, device manufacturer, drug manufacturer, device trade name, keyword, floating subheading word, candidate term word]	Publication date: 2013–2019 Filtered by: Humans
MEDLINE® via Ovid	
(Canada or “North America” or “United States” or “United States of America” or USA or Mexico or “United Mexican States” or “British Columbia” or Alberta or Manitoba or Saskatchewan or Ontario or Quebec or “Prince Edward Island” or PEI or “Nova Scotia” or “New Brunswick” or Newfoundland or Labrador or Yukon or Nunavut or “Northwest Territories” or NWT or Alabama or Alaska or Arizona or Arkansas or California or Colorado or Connecticut or Delaware or Florida or Georgia or Hawaii or Idaho or Illinois or Indiana or Iowa or Kansas or Kentucky or Louisiana or Maine or Maryland or Massachusetts or Michigan or Minnesota or Mississippi or Missouri or Montana or Nebraska or Nevada or “New Hampshire” or “New Jersey” or “New Mexico” or “New York” or “North Carolina” or “North Dakota” or Ohio or Oklahoma or Oregon or Pennsylvania or “Rhode Island” or “South Carolina” or “South Dakota” or Tennessee or Texas or Utah or Vermont or Virginia or Washington or “West Virginia” or Wisconsin or Wyoming or Aguascalientes or “Baja California” or Campeche or Chiapas or Chihuahua or Coahuila or Colima or Durango or Guanajuato or Guerrero or Hidalgo or Jalisco or Mexico or Michoacan or Morelos or Nayarit or “Nuevo Leon” or Oaxaca or Puebla or Queretaro or “Quintana Roo” or “San Luis Potosi” or Sinaloa or Sonora or Tabasco or Tamaulipas or Tlaxcala or Veracruz or Yucatan or Zacatecas).mp. [mp = title, abstract, original title, name of substance word, subject heading word, floating sub-heading word, keyword heading word, organism supplementary concept word, protocol supplementary concept word, rare disease supplementary concept word, unique identifier, synonyms] AND (“climate change” or weather or “atmospheric pressure” or “climatic change” or “global warming” or “environmental change” or “climate disaster” or “greenhouse effect” or “climate variability” or “climatic variability” or “carbon emission” or cold or cool or cooling or heat or humid* or ice or precipitation or rain* or season* or snow* or storm or temperature or warm or warming or wind or “ultraviolet radiation” or UV).mp. [mp = title, abstract, original title, name of substance word, subject heading word, floating sub-heading word, keyword heading word, organism supplementary concept word, protocol supplementary concept word, rare disease supplementary concept word, unique identifier, synonyms] AND (health or disease* or morbidity or mortality or wellbeing or illness* or wellness or infect* or death or injur* or mental* or emotion*).mp. [mp = title, abstract, original title, name of substance word, subject heading word, floating sub-heading word, keyword heading word, organism supplementary concept word, protocol supplementary concept word, rare disease supplementary concept word, unique identifier, synonyms]	Publication date: 2013–2019 Filtered by: Humans

## Appendix 2

Data extraction form

**Table:**

Data extraction category	Information extracted
Type of research	
Research Study	The article describes research with data collection and analysis.
Review using systematic methods	The article describes secondary research that clearly reported review methods.
Country*	
Canada	The article describes data collected in Canada.
United States of America	The article describes data collected in the USA.
Mexico	The article describes data collected in Mexico.
Non-North American Regions	The article describes data collected outside of North America. Note: To be included, the article also had to include data collected in Canada, USA, and/or Mexico.
Focus of study*	
Climate change impacts	The article describes research focused on the effects of climatic impacts in the goal, methods, and/or results section of the article.
Climate change adaptation	The article describes research focused on strategies/actions to deal with climate change impacts (e.g. flood evacuation plans for coastal communities) in the goal, methods, and/or results section of the article.
Climate change mitigation	The article describes research focused on strategies/actions to prevent climate change (e.g. reducing emissions) in the goal, methods, and/or results section of the article.
Weather variables*	
Temperature	The article describes research using data on temperature in the goal, methods, and/or results section of the article.
Precipitation	The article describes research using data on precipitation in the goal, methods, and/or results section of the article.
UV radiation	The article describes research using data on UV radiation in the goal, methods, and/or results section of the article.
Other	The article describes research using data on other climatic variables (e.g. humidity (includes relative humidity or index humidity), seasons, seasonality, changes in season, wind, El Nino/El Nina, etc. in the goal, methods, and/or results section of the article.
Climatic hazards*	
Heat events	The article describes research focused on heat events, including extreme heat, heat waves, in the goal, methods, and/or results section of the article.
Cold events	The article describes research focused on cold events, including extreme cold, blizzards, and winter storms, in the goal, methods, and/or results section of the article.
Air quality	The article describes research focused on air quality, including pollution, data on PPM, and greenhouse gas emissions, in the goal, methods, and/or results section of the article.
Drought	The article describes research focused on droughts in the goal, methods, and/or results section of the article.
Flooding	The article describes research focused on flooding in the goal, methods, and/or results section of the article.
Wildfires	The article describes research focused on forest and/or wildfires in the goal, methods, and/or results section of the article.
Hurricanes	The article describes research focused on hurricanes in the goal, methods, and/or results section of the article.
Wildlife changes	The article describes research focused on wildlife changes, including changes in vectors (e.g. ticks, mosquitoes), in the goal, methods, and/or results section of the article.
Vegetation changes	The article describes research focused on vegetation changes, including pollen changes, in the goal, methods, and/or results section of the article.
Freshwater	The article describes research focused on freshwater, including lake/river bodies and drinking water, in the goal, methods, and/or results section of the article.
Ocean conditions	The article describes research focused on ocean/sea conditions, including sea level rise, and ocean acidity/salinity/temperature, in the goal, methods, and/or results section of the article.
Ice extent/stability/duration	The article describes research focused on changes in ice extent/stability/duration, including sea ice and freshwater ice, in the goal, methods, and/or results section of the article.
Coastal erosion	The article describes research focused on coastal erosion in the goal, methods, and/or results section of the article.
Permafrost changes	The article describes research focused on permafrost changes in the goal, methods, and/or results section of the article.
Environmental hazards	The article describes research focused on environmental hazards occurring due to climatic hazards, including reduced crop production, sewage exposure, and fecal runoff, in the goal, methods, and/or results section of the article.
Health outcomes*	
Heat stress, morbidity, and/or mortality	The article describes research focused on heat stress, morbidity, and/or mortality, including heat stroke and heat stress, in the goal, methods, and/or results section of the article.
Respiratory outcomes	The article describes research focused on respiratory health, including asthma and COPD, in the goal, methods, and/or results section of the article.
Cardiovascular outcomes	The article describes research focused on cardiovascular disease, including heart attacks and stroke, in the goal, methods, and/or results section of the article.
Urinary outcomes	The article describes research focused on urinary diseases, including urinary tract infections and renal failure, in the goal, methods, and/or results section of the article.
Dermatologic concerns	The article describes research focused on dermatologic concerns, including sunburns and melanoma, in the goal, methods, and/or results section of the article.
Mental health and wellness	The article describes research focused on mental health and wellbeing, including suicide, psychoses, and emotional health, in the goal, methods, and/or results section of the article.
Fetal health, birth outcomes, and/or maternal health	The article describes research focused on fetal health, birth outcomes, and/or maternal health in the goal, methods, and/or results section of the article.
Cold exposure	The article describes research focused on cold exposures, including frostbite and cold-related morbidity/mortality, in the goal, methods, and/or results section of the article.
Allergies	The article describes research focused on allergies in the goal, methods, and/or results section of the article.
Nutrition	The article describes research focused on nutrition, including food security, nutrition transition, and nutrient deficiency, in the goal, methods, and/or results section of the article.
Waterborne disease	The article describes research focused on waterborne disease in the goal, methods, and/or results section of the article.
Foodborne disease	The article describes research focused on foodborne disease in the goal, methods, and/or results section of the article.
Vectorborne disease	The article describes research focused on vectorborne disease in the goal, methods, and/or results section of the article.
Injuries	The article describes research focused on injuries, including fractures and accidents, in the goal, methods, and/or results section of the article.
General morbidity and/or mortality	The article describes research focused on general morbidity and/or mortality that generally references human health in the goal, methods, and/or results section of the article.
Other outcomes	The article describes research focused on other health outcomes, including diabetes and electrolyte imbalance, in the goal, methods, and/or results section of the article.
Social variables*	
Access to healthcare	The article describes research using data about access to healthcare in the goal, methods, and/or results section of the article.
Sex and/or gender	The article describes research using data on gender and/or sex in the goal, methods, and/or results section of the article.
Age	The article describes research using data on age in the goal, methods, and/or results section of the article.
Income	The article describes research using data on income in the goal, methods, and/or results section of the article.
Livelihood	The article describes research using data on livelihoods, including employment and occupation, in the goal, methods, and/or results section of the article.
Ethnicity	The article describes research using data on ethnicity and/or race in the goal, methods, and/or results section of the article.
Culture	The article describes research using data on culture in the goal, methods, and/or results section of the article.
Indigenous Peoples	The article describes research focused on Indigenous Peoples in the goal, methods, and/or results section of the article.
Rural communities	The article describes research explicitly focused on rural/remote communities in the goal, methods, and/or results section of the article. The authors had to describe the location as “rural”, “remote”, or other similar terms to fit this classification.
Urban communities	The article describes research explicitly focused on urban communities, including cities and metropolitan areas, in the goal, methods, and/or results section of the article. The authors had to describe the location as “urban”, “city”, “metropolitan”, or similar terms to fit this classification.
Coastal communities	The article describes research focused on coastal communities in the goal, methods, and/or results section of the article. These communities could also be defined as urban or rural/remote. The authors had to explicitly describe the location as “coastal” or similar terms to fit this classification.
Residence location	The article describes research using data on location of residence, including zipcode/postal code, and neighbourhood, in the goal, methods, and/or results section of the article.
Education	The article describes research using data on level of education in the goal, methods, and/or results section of the article.
Housing	The article describes research using data on housing, including size, age, number of windows, and air conditioning, in the goal, methods, and/or results section of the article.
Future projections*	
Qualitative projections	The article describes research that used future projections through qualitative methods in the goal, methods, and/or results section of the article. This includes quotes or narratives of research participants qualitatively describing future scenarios.
Quantitative projections	The article describes research that used future projections through quantitative methods in the goal, methods, and/or results section of the article. This includes quantitative models projecting risks in the future.
No future projections or scenarios	The article does not describe research that used projections or future scenarios in the goal, methods, and/or results section of the article.

*Categories were not mutually exclusive; that is, more than one category could be selected

## Abbreviations

CERQual: Confidence in the Evidence from Reviews of Qualitative Research
IPCC: Intergovernmental Panel on Climate Change
MMAT: Mixed Methods Appraisal Tool
PPM: Parts per million
PRISMA: Preferred Reporting Items for Systematic review and Meta-Analyses
PRISMA-P: Preferred Reporting Items for Systematic review and Meta-Analyses, Protocol Extension
USA: United States of America
UV: Ultraviolet
