# Supplementary figures and images for: Climate change and health in North America: literature review protocol
Source: Syst Rev. 2021 Jan 4;10:3. doi: 10.1186/s13643-020-01543-y (PMC7780400; doi:10.1186/s13643-020-01543-y)

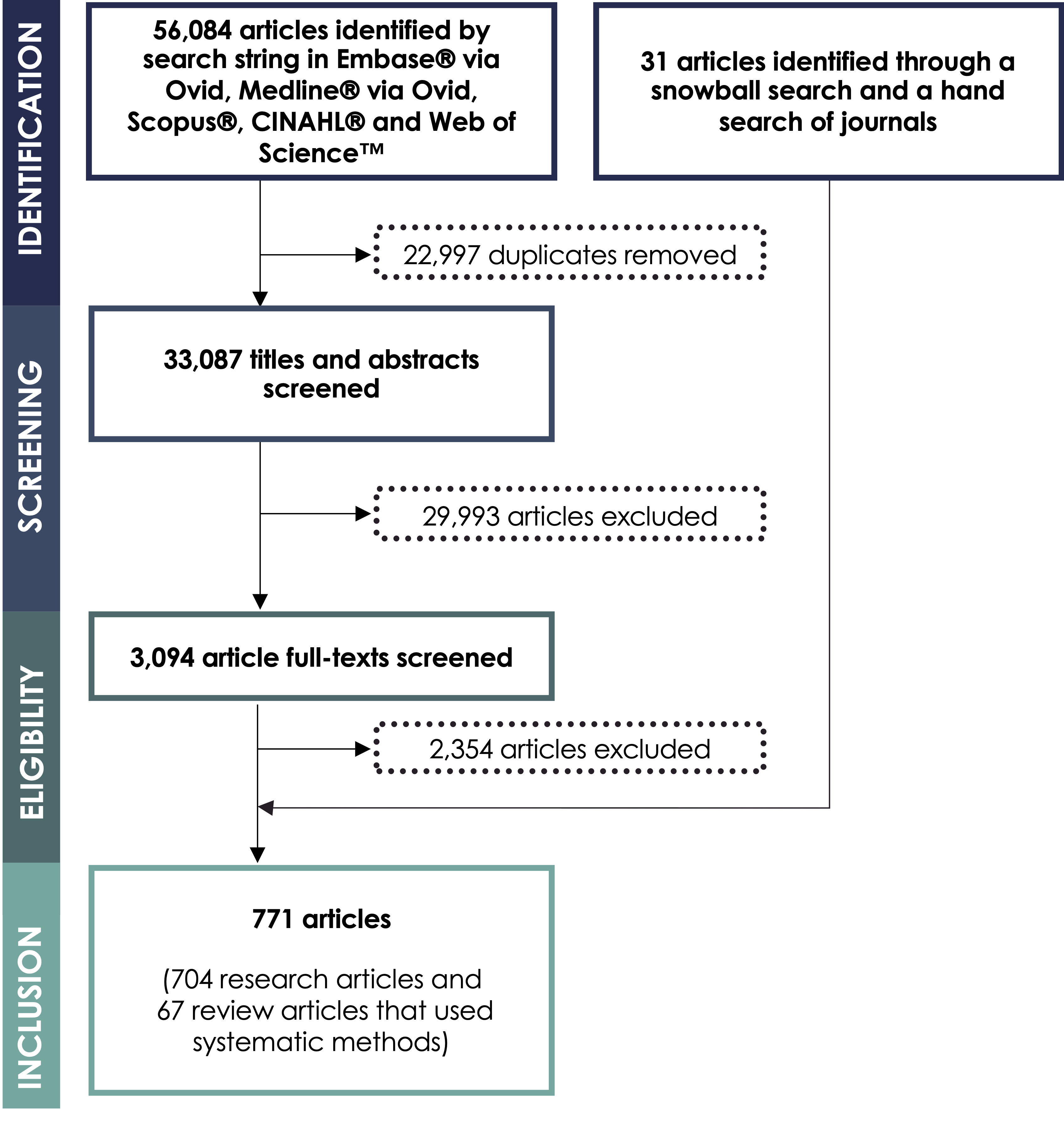

Supplement: Supplementary file 1 — Additional file 1. [file 13643_2020_1543_MOESM1_ESM.jpg]
